# High‐throughput sequencing and graph‐based cluster analysis facilitate microsatellite development from a highly complex genome

**DOI:** 10.1002/ece3.2305

**Published:** 2016-07-22

**Authors:** Abhijeet B. Shah, Holger Schielzeth, Andreas Albersmeier, Joern Kalinowski, Joseph I. Hoffman

**Affiliations:** ^1^ Department of Animal BehaviourBielefeld UniversityPostfach 10013133501BielefeldGermany; ^2^ Department of Evolutionary BiologyBielefeld UniversityMorgenbreede 4533615BielefeldGermany; ^3^ Department of Population Ecology, Institute of EcologyFriedrich Schiller University Jena, Dornburger Str. 15907743JenaGermany; ^4^ Center for BiotechnologyUniversitätsstraße 2533615BielefeldGermany

**Keywords:** Acrididae, genetic marker development, *Gomphocerus sibiricus*, high‐throughput sequencing, microsatellite, Orthoptera, transposable elements

## Abstract

Despite recent advances in high‐throughput sequencing, difficulties are often encountered when developing microsatellites for species with large and complex genomes. This probably reflects the close association in many species of microsatellites with cryptic repetitive elements. We therefore developed a novel approach for isolating polymorphic microsatellites from the club‐legged grasshopper (*Gomphocerus sibiricus*), an emerging quantitative genetic and behavioral model system. Whole genome shotgun Illumina MiSeq sequencing was used to generate over three million 300 bp paired‐end reads, of which 67.75% were grouped into 40,548 clusters within RepeatExplorer. Annotations of the top 468 clusters, which represent 60.5% of the reads, revealed homology to satellite DNA and a variety of transposable elements. Evaluating 96 primer pairs in eight wild‐caught individuals, we found that primers mined from singleton reads were six times more likely to amplify a single polymorphic microsatellite locus than primers mined from clusters. Our study provides experimental evidence in support of the notion that microsatellites associated with repetitive elements are less likely to successfully amplify. It also reveals how advances in high‐throughput sequencing and graph‐based repetitive DNA analysis can be leveraged to isolate polymorphic microsatellites from complex genomes.

## Introduction

Although SNPs are increasing in popularity, microsatellites remain an important class of molecular marker due to their low cost and flexibility (Schlotterer 2004). In particular, high levels of polymorphism make microsatellites ideally suited to parentage analysis, particularly for breeding designs involving large numbers of offspring but relatively few candidate parents (Jones and Ardren 2003). In these situations, a handful of highly polymorphic markers can provide a straightforward and cost effective means of constructing pedigree relationships. High levels of polymorphism also make microsatellites suitable for quantifying levels of inbreeding, at least in some species where moderate numbers of microsatellites have been found to outperform substantial panels of SNPs (Forstmeier et al. 2012).

Arguably, one of the greatest disadvantages of microsatellites is the laborious, time consuming and expensive process of developing them in nonmodel species, which until recently required the construction of enriched genomic libraries followed by cloning, hybridization to detect the positive clones and Sanger sequencing (Zane et al. 2002). However, the advent of high‐throughput sequencing approaches, initially Roche 454 but later Illumina sequencing, has simplified the discovery process and now allows many thousands of microsatellite containing sequences to be isolated from virtually any organism (Abdelkrim et al. 2009; Santana et al. 2009; Rico et al. 2013).

Despite the growing ease and popularity of mining for microsatellites *in silico*, a number of issues remain unresolved. In particular, it is still necessary to design oligonucleotide primers from microsatellite flanking sequences and test these for polymorphism in a representative sample of individuals, a process that is both time consuming and costly. Moreover, success rates vary considerably among species (McInerney et al. 2011) and it is not unusual for a significant proportion of primers either to fail to generate interpretable PCR products or to amplify microsatellites that are monomorphic, show evidence of null alleles, or which are inconsistent with a single Mendelian locus (David et al. 2003).

Species with large and complex genomes, including many plants and invertebrates are particularly problematic (Garner 2002). This is because cryptic repetitive elements including transposable elements are disproportionately abundant in large genomes, reaching frequencies as high as 80% in some grasses (Feschotte et al. 2002). Moreover, microsatellites are often not randomly distributed throughout genomes, but instead tend to be preferentially associated with transposable elements such as short interspersed repeats (SINEs) and long interspersed elements (LINEs) (Ramsay et al. 1999). It has even been suggested that repetitive elements could be involved in the genesis and propagation of microsatellites (Arcot et al. 1995; Nadir et al. 1996; Wilder and Hollocher 2001) although it is also possible that transposable element insertion could be favored at sites containing pre‐existing microsatellites (Ellegren 2004). Regardless of their exact provenance, microsatellites associated with repetitive elements will exist in multiple copies in the genome where they will have similar or near identical flanking sequences (Zhang 2004). This has been invoked as an explanation for the poor success rates (ranging from zero to around twenty percent) of efforts to develop microsatellites in species as diverse as Norway spruce (Pfeiffer et al. 1997), butterflies (Meglecz et al. 2004), squat lobsters (Bailie et al. 2010), and parasitic nematodes (Grillo et al. 2006).

One way to circumvent this problem is to develop microsatellites from expressed sequence tag libraries (Grillo et al. 2006) or other transcriptomic resources (Blondin et al. 2013), as cryptic elements should be less abundant in selectively constrained regions of the genome. As long as multiple individuals are used for sequencing the transcriptome, this additionally allows microsatellites to be screened for polymorphism *in silico* (Hoffman and Nichols 2011). However, generating a transcriptome is less straightforward than shotgun genome sequencing and also tends to yield microsatellites with lower average levels of polymorphism (Dufresnes et al. 2014). Thus, an attractive alternative would be to identify and remove repetitive sequences from a pool of genomic sequence reads, allowing development efforts to be focused on single‐copy microsatellites.

Orthopterans are a group of organisms for which microsatellite development can be particularly problematic. Many species of grasshoppers and locusts are famous for their large genomes (Gregory 2015), like the Acridid grasshoppers, which have haploid genome sizes of around 6–16 Gb (Gregory 2015). A further peculiarity of grasshoppers is the frequent occurrence of facultative (supernumerary) chromosomes that further increase the amount of DNA per cell (Palestis et al. 2004) and hence the potential for primers to bind at multiple sites. The 6.5 Gb genome of the migratory locust *Locusta migratoria* has recently been sequenced and has been found to contain about 60% repetitive elements, of which DNA transposons and LINE retrotransposons are the most abundant (Wang et al. 2014). In addition, a recent study of two other grasshopper species showed by fluorescent *in situ* hybridization that microsatellites are strongly associated with repetitive elements including histone gene spacers, ribosomal DNA intergenic spacers and transposable elements (Ruiz‐Ruano et al. 2014).

The number of published microsatellites for Acridid grasshoppers is rather low and these all required the screening of very large numbers of candidate loci (Ustinova et al. 2006; Grace et al. 2009; Chapuis et al. 2012; Keller et al. 2012; Blondin et al. 2013). The club‐legged grasshopper, *Gomphocerus sibiricus*, that we study here is an Acridid grasshopper with a sizable genome of around 8.7 Gb (Gregory 2015) and a high prevalence of supernumerary chromosomes (López‐Fernández et al. 1986). This species is a valuable model system for studying the evolution sexual ornamentation and the long‐term maintenance of color polymorphisms in natural populations (Valverde and Schielzeth. 2015). Fitness assays under competitive conditions in the field and in the laboratory, quantitative genetic studies and inbreeding studies in relation to sexual ornamentation all require genetic markers, yet none are currently available.

Here, we developed an approach for isolating polymorphic microsatellites from complex genomes based on shotgun Illumina MiSeq sequencing and downstream bioinformatic analysis. Specifically, our pipeline incorporates RepeatExplorer (Novak et al. 2013), a collection of software tools that implements graph‐based clustering of unassembled sequence reads in order to identify repetitive elements *de novo*. We then exclude reads associated with clusters of repetitive DNA, identify microsatellite motifs within the remaining singletons using Pal_finder (Castoe et al. 2012) and design primers within Pal_finder using Primer3 (Untergasser et al. 2012). We demonstrate experimentally that primers designed in this way have a significantly greater likelihood of generating clearly interpretable and polymorphic PCR products than primers associated with clusters.

## Materials and Methods

### Sample collection and preparation for high‐throughput sequencing

Grasshoppers were collected near Sierre Valais, Switzerland (46°20′N, 7°30′E) and stored in 70% ethanol at −20°C. Genomic DNA was later extracted from the hind leg using a standard chloroform‐isoamyl alcohol protocol (Sambrook et al. 1989).

### High‐throughput sequencing

Illumina sequencing of five individuals (three males and two females) was conducted at the Center for Biotechnology (CeBiTec) at Bielefeld University. Libraries were prepared with the Nextera DNA Sample Preparation Kit (Illumina, Little Chesterford, UK) according to the manufacturer's instructions. The DNA was then run on a 1.5% agarose gel and fragments in the size range 600–1000 bp were extracted with the Qiagen MinElute Gel Extraction Kit (Qiagen, Hilden, Germany). Fragment sizes were checked using a High Sensitivity DNA Chip on the Agilent 2100 Bioanalyzer (Agilent, Waldbronn, Germany). Quantification was performed using the Quant‐iT Picogreen^®^ dsDNA Assay Kit (Life Technologies, Darmstadt, Germany). The libraries were then sequenced on an Illumina MiSeq sequencer using a MiSeq^®^ Reagent Kit v3 (600 cycles; Illumina) to generate 301 bp paired‐end reads. FastQ files were generated automatically by the software MiSeq Reporter(version 2.5.1.3: Illumina Inc, 5200 Illumina Way, 92122 San Diego, CA, USA). Analysis within FastQC (Andrews) indicated that the reads were of high quality.

### Graph‐based repeat characterization and identification

Graph‐based clustering and characterization of repetitive sequences was conducted using RepeatExplorer (pipeline version 198+ stable) (Novak et al. 2013) following the developers recommendations (http://repeatexplorer.umbr.cas.cz). Reads that were identified as singletons were retained for microsatellite mining, whereas reads that were assigned to clusters by RepeatExplorer were discarded.

### Trinity assembly

As an alternative to using RepeatExplorer to assemble the repetitive DNA elements, we also tested the utility of the *de novo* assembly program Trinity version 2.1.1 (Haas et al. 2013). We first merged the forward and reverse reads within Pear version 0.9.8 (Zhang et al. 2014) using the default parameters. We then assembled the resulting reads within Trinity using the default parameters.

### Microsatellite mining and primer design

The resulting singleton reads were mined for microsatellites (more specifically, potentially amplifiable loci or PALs) using the script Pal_finder version 0.02.04 (Castoe et al. 2012). For simplicity and to avoid the issue of primers spanning forward and reverse reads, only the forward reads were used. The reads were interrogated for di‐, tri‐, tetra‐, penta‐ and hexanucleotides containing at least eight tandem repeats. Within PAL_FINDER version 0.0.2.04, we then used Primer3 version 2.0.0 (Untergasser et al. 2012) to design primers for the target loci. Default parameters were used except for the PCR product size range, which was set to 100–250 bp, and the annealing temperature range, which was set to 55–65°C.

### Filtering criteria of PALs

The output from PAL_FINDER was filtered to remove PALs for which primers could not be designed, PALs that occurred in five or more different reads, and PALs where the primer sequences had phred quality scores lower than 29 for at least 95% of the forward and reverse primer bases. As a last step to exclude any loci with multiple copies, we then BLASTed the remaining PALs against all of the forward reads and excluded all PALs with five or more BLAST hits.

### *In vitro* testing of PALs

We attempted to obtain a representative sample of the filtered PALs by randomly selecting 18 of the 484 dinucleotide repeats, 18 of the 78 trinucleotide repeats and all 12 of the tetranucleotide repeats identified above. PCR primer pairs for these loci were tested for polymorphism in eight wild‐caught individuals. Each locus was fluorescently labeled using the M13‐tail approach (Schuelke 2000) and PCR amplified using a Type It kit (Qiagen). The following PCR profile was used: one cycle of 30 sec at 95°C; 25 cycles of 45 sec at 94°C, 45 sec at 60°C and 45 sec at 72°C; 23 cycles of 30 sec at 94°C, 45 sec at 53°C and 45 sec at 72°C; and one final cycle of 10 min at 72°C. PCR products were resolved by electrophoresis on an ABI 3730xl capillary sequencer.

### Pipeline validation

To test whether PALs mined from singletons have greater amplification success than PALs residing within clusters, we additionally evaluated 21 dinucleotide, 21 trinucleotide and six tetranucleotide repeats mined from reads associated with randomly selected clusters. On each PCR plate, we included two positive controls comprising polymorphic loci from the first round of testing.

### Multiplexing

We selected 20 polymorphic microsatellites from the first round of testing for incorporation into two PCR multiplexes. These were then used to genotype the original eight individuals of the initial screen plus 32 additional individuals from the same population. For each multiplex reaction, we used a Type It kit (Qiagen) with the following PCR conditions: one cycle of 5 min at 95°C; 25 cycles of 30 sec at 94°C, 90 sec at 60°C, and 60 sec at 72°C; followed by one final cycle of 30 min at 60°C. PCR products were resolved by electrophoresis on an ABI 3730xl capillary sequencer.

### Scoring and data analysis

Allele sizes were scored using the program GeneMarker version 2.6_2 (Softgenetics). To ensure high genotype quality, all traces were manually inspected and any incorrect calls were adjusted accordingly. Genepop on the web (Raymond and Rousset 1995) was then used to calculate the observed and expected heterozygosities and to test for deviations from Hardy–Weinberg equilibrium (HWE), specifying a dememorization number of 10,000, 1000 batches and 10,000 iterations per batch.

### Ethics statement

All the field samples were taken from individual‐rich populations and in accordance with institutional, national, or international legislation and guidelines. No specific permissions were required for the collection of this neither endangered nor protected species outside protected areas.

## Results

**Figure 1:**
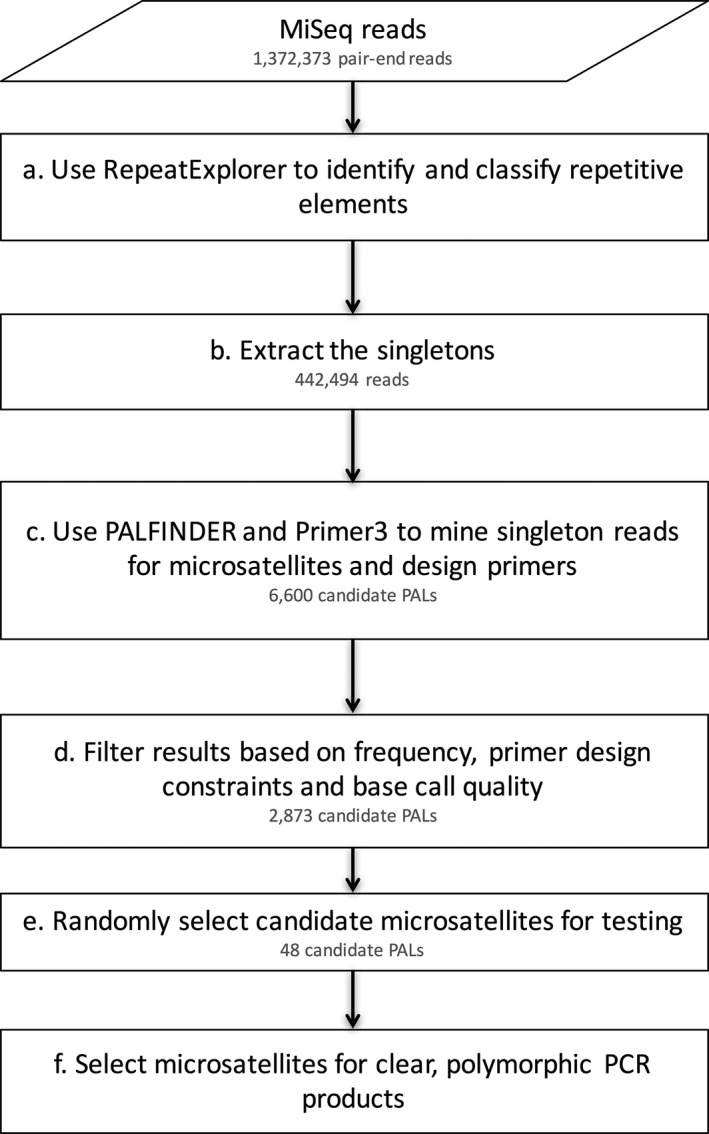
Flowchart detailing the bioinformatic pipeline used to identify polymorphic microsatellites in the club‐legged grasshopper, *Gomphocerus sibricus*.

**Figure 2:**
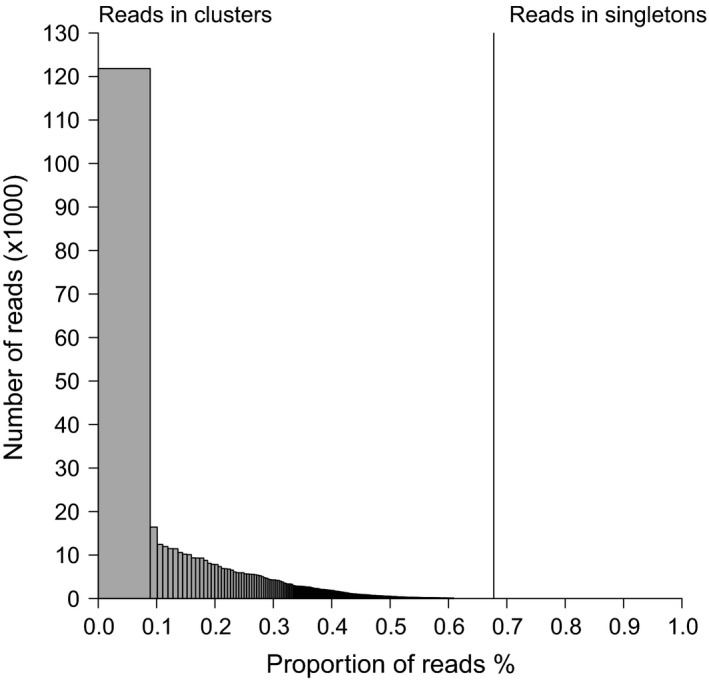
Results of RepeatExplorer analysis showing the numbers of reads classified as clusters or singletons.

**Figure 3:**
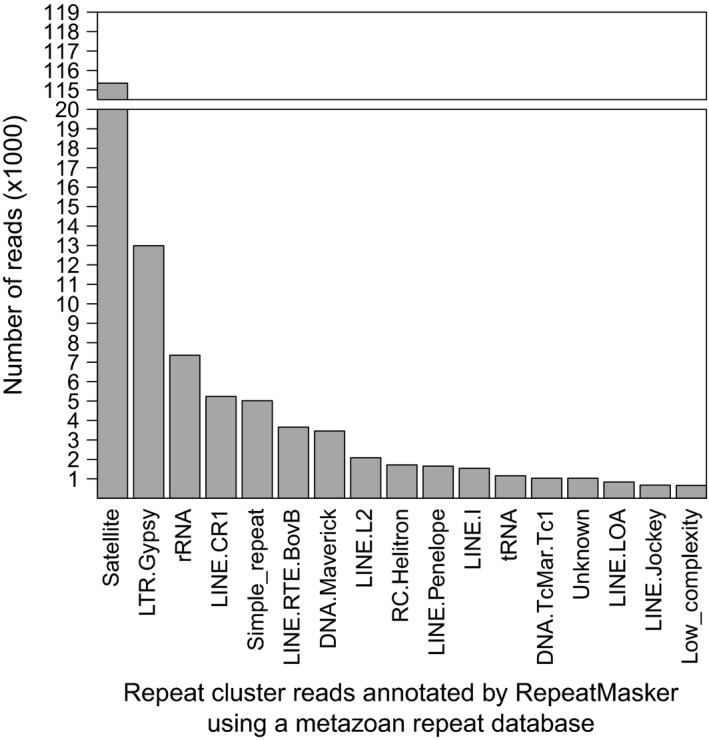
A summary of the RepeatMasker analysis showing the number of reads annotated for repeative elements with at least 550 reads using a metazoan repeat database.

Three male and two female wild‐caught *Gomphocerus sibricus* individuals were sequenced on part of an Illumina MiSeq run, resulting in 3,197,707 paired‐end reads totaling approximately 1.92 Gb (approximate average coverage = 0.05 for a genome of 8.7 Gb (Gregory 2015)). These data were subjected to the bioinformatic workflow outlined in Figure 1. First, we used RepeatExplorer to identify and classify repetitive elements (Step *a* of the pipeline in Fig. 1). This program analyzed a subsample of the sequence data comprising 1,372,373 reads. Of these, 929,879 (67.75%) were grouped into 40,548 clusters (Fig. 2), whereas the remaining 442,494 reads were characterized as “singletons”. The top 468 clusters, which account for 60.5% of the reads containing repetitive elements were annotated by RepeatExplorer, revealing that a large proportion show similarity to cryptic repetitive DNA elements (Fig. 3). Consequently, in order to improve the probability of success, we discarded reads associated with clusters, leaving only the singleton reads (Step *b*, Fig. 1), from which we mined microsatellites (Step *c*, Fig. 1).

### Microsatellite mining and primer design

PAL_FINDER identified 6,600 PALs. Of these, primers could be designed for 2,873 (43.5%) using the parameters specified in the Materials and methods. PALs with primer sequences that occurred in five or more reads and/or which had phred scores below 29 were removed, leaving 987 PALs (Step *d*, Fig. 1). These carried between eight and 48 tandem repeats (mean = 14.04). We selected 48 of these for *in vitro* testing (Step *e*, Fig. 1).

### In vitro verification

Of the 48 primer pairs, 30 (62.5%) yielded clear PCR products that could be discriminated as either polymorphic (*n *=* *29 loci, of which 17 were clearly interpretable and amplified in at least six of the eight individuals) or monomorphic (*n *=* *1 locus) in a sample of eight unrelated *G. sibricus* individuals. The high quality polymorphic loci carried between two and 11 alleles each (mean = five alleles) and observed heterozygosity ranged from 0.125 to 1.0 (Table 1). Four of these loci deviated significantly from HWE (Table 1), although not after false discovery rate (FDR) correction for multiple tests. Of those 18 loci that failed to amplify PCR products resembling microsatellites, six amplified multiple bands that looked similar to amplified fragment length polymorphisms (AFLPs), and the remaining failed to generate any discernable products.

**Table 1:** *In vitro* verification of the primer pairs. Shown are the polymorphism characteristics of 17 microsatellite loci that amplified clearly interpretable and polymorphic PCR products in eight unrelated *Gomphocerus sibricus* individuals

Locus	Repeat motif	Tandem repeats	Forward primer	Reverse primer	Number of alleles	*H*_O_	*H*_E_	HWE *P*‐value
Gsib01	TC	16	AGAGGGAGACAGATAGACGGC	TTCCACACTTTTAAGACTGAATGC	10	1.00	0.93	1.00
Gsib02	TC	10	CTGATTCACAGATAGGGGCG	GTCCATATCCTCCTCCCTCC	5	0.50	0.82	0.09
Gsib07	AC	8	ACACACAACTGCAAACTCCG	TCTTCAGAAAAGATCTCTCCCC	11	1.00	0.93	1.00
Gsib08	TC	8	AGAGACCACAGGCAGAGAGC	CCCTTTATTGATCGCAAAGC	2	0.17	0.53	0.15
Gsib13	TC	21	TGAAATCCATGTAGCATCGC	CGGACTTCAACGAAGATTCC	9	0.88	0.12	0.88
Gsib16	TC	8	TGTGCGATCTTACTCGACCC	GGCCACTTCTTTGTCAGAGC	6	0.38	0.86	0.01
Gsib18	TC	11	AAGGGAGAAGGAAGACGTGC	GAGAAACATGATGTCGACCG	8	0.75	0.91	0.08
Gsib19	ATC	10	TCTATGCTCCAGACGGAACG	CAGACATGAAGCCAAAACCC	6	0.88	0.82	0.57
Gsib21	ATC	9	ACACAAAATATTCCGTGCCC	GACTTACACCAGGTAGGGCG	3	0.50	0.66	0.38
Gsib24	ATC	9	AGTCTAACGGCCAGAAATGC	TAGTTTTGGCGAAGGAGTCG	3	0.75	0.67	1.00
Gsib28	ATT	8	ATGTTCATGGTGACAATGCC	CCCCTCACAGGTTATCTTTGC	2	0.25	0.50	0.22
Gsib29	ATT	8	TCTAGAACCTTTGGTCTGTTGC	ACGAATGTCCCAAGAACAGG	3	0.12	0.61	0.01
Gsib32	TCC	10	CTACCTTCCTCCTATCGCCC	ATGTGGTTCCCTGTTTCTGC	6	0.75	0.84	0.38
Gsib35	ATC	10	TATGCTGCAATATGCTTGGC	TCCTCACAAGTGCAGAATGC	3	0.50	0.42	1.00
Gsib42	ATAC	9	GAGGCTGTAGCCATTTCTCG	GTCTTCACTTCCCATGAGGC	3	0.17	0.71	0.01
Gsib45	AAAG	8	CAAGGCCACAGTTAAGGAGG	AATGTCTGTGAAATATTACGTGCC	3	0.62	0.67	0.66
Gsib46	AATC	9	TATTGCCTCTGAATCTGCCC	ATATAGCTGTCCTCAGCGCC	2	1.00	0.53	0.03

HWE, Hardy–Weinberg equilibrium.

### Results of the trinity analysis

Finally, as an alternative to using RepeatExplorer to assemble repetitive DNA elements, we also carried out a *de novo* assembly of the raw reads using Trinity (Haas et al. 2013). This resulted in a total of 153,997 contigs with an N50 value of 677. We then BLASTed the 1223 PALs mined from clusters identified by RepeatExplorer and the 987 PALs mined from singletons against the Trinity assembly using a minimum identity match of 95% and only retaining the top hit. The majority of the PALs mined from RepeatExplorer clusters (908, 74.2%) revealed top hits to the Trinity assembly, while almost none of the PALs residing within singletons showed sequence homology to the Trinity contigs. This indicates that Trinity preferentially assembled the repetitive elements and hence could be used as an alternative to RepeatExplorer.

### Pipeline verification

Our microsatellite discovery pipeline is based on the premise that PALs residing within singleton reads should amplify more successfully than PALs residing within clusters. To test this prediction empirically, we evaluated a “control” set of 48 PALs mined from reads assigned to clusters of repetitive elements (see Materials and Methods for details). Only five of these loci (10.4%) yielded polymorphic PCR products consistent with the amplification of a single locus. A further two microsatellites were polymorphic but appear to be duplicated as individuals carry up to four alleles each. Ten additional loci were monomorphic and the remaining 31 loci failed to generate interpretable banding patterns. Multiple peaks, often resembling AFLP profiles, were observed in 27 of the latter, while four failed to generate any PCR products. The difference in the success rate of PALs within singletons and clusters, defined as the proportion generating polymorphic banding patterns consistent with a single locus, was highly significant (29/48 versus 5/48; two‐tailed binomial proportions test, *χ* ^2^ = 24.1, df = 1, *P *<* *0.0001).

### Multiplexing

Finally, we selected twenty loci for inclusion in two multiplexes and genotyped these in a larger panel of 40 unrelated individuals. One locus did not amplify polymorphic PCR products and a further ten loci deviated significantly from HWE after FDR correction (Table 2). The remaining nine loci were clearly interpretable and did not deviate from HWE.

**Table 2:** Polymorphism characteristics of 20 microsatellite loci that were multiplexed and amplified in 40 unrelated *Gomphocerus sibricus* individuals. The initial PCR mixes (1 and 2) were modified to minimize interference between loci, resulting in mixes 1a, 1b, 2a, and 2b. * denotes HWE tests that remained significant after table‐wide false discovery rate correction for multiple statistical testing

Locus	Mix						Dye	Size range (bp)	Number of alleles	H_O_	H_E_	HWE *P*‐value
	1	1a	1b	2	2a	2b						
Gsib01	x	x					FAM	99–151	22	0.53	0.93	<0.0001*
Gsib02	x	x	x				PET	244–255	10	0.64	0.82	0.0517
Gsib03	x						FAM	280–284	2	0.11	0.10	1.00
Gsib07				x	x		FAM	93–132	19	0.64	0.89	<0.0001*
Gsib09				x	x	x	NED	182	1	1.00	1.00	NA
Gsib13	x	x	x				PET	167–232	17	0.38	0.90	<0.0001*
Gsib14	x	x	x				VIC	119–129	5	0.21	0.71	<0.0001*
Gsib16					x		FAM	188–232	14	0.72	0.86	0.1961
Gsib17	x		x				FAM	180–212	11	0.24	0.68	<0.0001*
Gsib18				x		x	FAM	162–223	22	0.74	0.93	<0.0001*
Gsib19	x						FAM	236–276	12	0.74	0.91	0.0304
Gsib21	x	x	x				VIC	171–186	6	0.36	0.68	<0.0001*
Gsib23		x					FAM	232–253	5	0.29	0.74	0.0001*
Gsib24				x	x	x	VIC	157–169	3	0.44	0.52	0.2274
Gsib28				x	x	x	NED	223–235	5	0.37	0.53	0.0937
Gsib29				x	x	x	PET	234–265	5	0.11	0.61	<0.0001*
Gsib32				x	x	x	VIC	202–230	11	0.67	0.86	0.029
Gsib35	x	x	x				VIC	204–226	3	0.38	0.32	0.706
Gsib36				x	x	x	PET	154–170	8	0.28	0.80	<0.0001*
Gsib45	x	x	x				NED	220–232	5	0.63	0.67	0.2345

HWE, Hardy–Weinberg equilibrium; H_O_, observed heterozygosity; H_E_, expected heterozygosity.

## Discussion

Poor microsatellite amplification success is often associated with the occurrence of cryptic repetitive elements (Pfeiffer et al. 1997; Grillo et al. 2006; Bailie et al. 2010; McInerney et al. 2011). A number of studies reached this conclusion on the basis of *post hoc* analyses of flanking sequence similarities revealed by all‐against‐all BLAST analysis (Meglecz et al. 2004; McInerney et al. 2011) and through comparison to the Repbase database of known transposable elements (McInerney et al. 2011). Our approach also exploits information on sequence similarity and homology to the Repbase database, but this time through graph‐based cluster analysis implemented within RepeatExplorer. However, it differs from previous approaches in two ways. First, we exploited high‐throughput sequencing to generate millions of reads, providing greater resolution of the composition of repetitive elements in the grasshopper genome, and second, we conducted the bioinformatic analysis prior to primer design and testing, allowing us to focus on single‐copy loci.

### Repetitive elements in the club‐legged grasshopper genome

Ours is not the first study to assign microsatellite flanking sequences to different families of repetitive element (Bailie et al. 2010; McInerney et al. 2011), although the use of high‐throughput sequencing allowed us to scale up from a few hundred Sanger sequences to over three million reads of similar length. By sequencing a library that was not enriched for microsatellite motifs, we could additionally obtain a tentative estimate of the overall proportion of genomic sequences containing repetitive elements. We found that over 67.8% of reads could be grouped into clusters, the top 468 of which accounted for approximately 60.5% of the sequence data. The fraction of repetitive elements is in close agreement with the migratory locust (Wang et al. 2014). However, the club‐legged grasshopper is unusual in that a single repeat class cluster dominates much more strongly than in other species with large genomes, including animals and plants (Piednoel et al. 2012; Garcia et al. 2015).

In our sample, the most abundant RepeatMasker hit was satellite DNA (see Fig. 3). This is not necessarily surprising as a previous study by Rafferty and Fletcher (Rafferty and Fletcher 1992) found that around 30% of the genome of *Stauroderus scalaris*, another member of the Gomphocerinae grasshopper family, also comprises satellite DNA. With limited data available on other Orthopteran species, we can only speculate as to how and to what extent the distribution and composition of repetitive elements differs among related taxa. The closest relative with genomic resources available, the migratory locust *Locust migratoria*, differs considerably in the composition of repetitive elements, a significant portion of which are DNA transposons and LINE retrotransposons. However, this might not be too surprising because the two species are divergent by approximately 57 million years (Song et al. online early) and even their genome sizes differ considerably (6.5 Gb versus 8.7 Gb, a 33% difference). Exploring how different classes of repetitive element may have invaded the genomes of different Orthopteran species now seems feasible given that more than one species could be pooled onto a single MiSeq run, providing a fertile avenue for future research.

### Microsatellite development success

We found that microsatellites developed from singleton reads had a six‐fold higher success rate, defined by the proportion of loci amplifying polymorphic products consistent with a single locus, relative to a set of microsatellites mined from reads associated with clusters. This supports a previous study of Norway spruce, which found that primer pairs amplifying a single polymorphic microsatellite were largely restricted to unique clone sequences that lacked repetitive DNA (Pfeiffer et al. 1997). Causes of microsatellite failure in our study included (1) PCR amplification failure, which resulted either in no discernible product or in a small number of nonspecific bands; (2) the amplification of monomorphic bands resembling microsatellite alleles; or (3) the amplification of more than one locus, indicated by the presence of up to four alleles within an individual. All of these patterns have been observed in similar studies of other species with complex genomes (Pfeiffer et al. 1997; Bailie et al. 2010; McInerney et al. 2011).

Elsewhere, microsatellites developed from a nematode species with an apparently complex genome were found to carry unusually high frequencies of nonamplifying or “null” alleles, indicated by the presence of multiple apparently homozygous non‐amplifying individuals (Grillo et al. 2006). As null alleles are caused by polymorphisms within the primer binding sites, these authors concluded that the species in question probably has very high levels of sequence polymorphism, reflecting the vast effective population sizes of many nematodes. It is for this reason that we selected twenty loci for inclusion in two mastermixes, which we then used to genotype a larger panel of 40 individuals. Having done this, we found that a considerable proportion of the loci did not conform to HWE in the larger sample. As the majority of these loci showed heterozygote deficiency, we conclude that null alleles may also be relatively common in club‐legged grasshoppers. Nevertheless, nine of the loci conformed to HWE, suggesting that with our approach it is eminently feasible to generate a panel of microsatellites large enough for most purposes.

### Possible alternatives to RepeatExplorer analysis

In this paper, we focused on using RepeatExplorer to *de novo* assemble and annotate repetitive elements. In principle, however, alternative assembly programs might be used to similar effect. To test this, we also *de novo* assembled our data using Trinity and then looked for overlap between PALs identified in the previous analysis as residing within clusters and singletons, respectively. We found that PALs residing within the clusters identified by RepeatExplorer predominantly mapped to the Trinity assembly. By implication, the Trinity assembly must be enriched for repetitive elements in the same way as the RepeatExplorer clusters, and hence it appears that both approaches could be useful for screening out PALs residing within repetitive elements. It would be interesting in the future to test whether such approaches bring similar benefits in other species and to further explore the merits of other approaches for *de novo* repeat discovery and sequence assembly.

## Conclusions

We used massively parallel sequencing together with graph‐based clustering and annotation to develop polymorphic microsatellites for the club‐legged grasshopper, an emerging quantitative genetic model system. Our study not only sheds light on the composition of the repetitive fraction of this species genome, but also demonstrates the potential of *in silico* filtering to dramatically improve the success of microsatellite development efforts.

## Conflict of Interest

None declared.

## Data Accessibility

The raw read sequences used in this analysis have been deposited in the short read archive with BioProject ID: PRJNA321244. Details of the microsatellites are provided in the Dryad data repository with the DOI: 10.5061/dryad.23r6v.
